# Low and high postpubertal ethanol use: damage on adulthood reproduction and offspring

**DOI:** 10.1530/RAF-22-0009

**Published:** 2022-07-28

**Authors:** Vanessa Caroline Fioravante, Alana Rezende Godoi, Victória Mokarzel de Barros Camargo, Patricia Fernanda Felipe Pinheiro, Marcelo Martinez, Carlos Roberto Padovani, Francisco Eduardo Martinez

**Affiliations:** 1 Department of Structural and Functional Biology, Institute of Biosciences of Botucatu (IBB), UNESP – Univ Estadual Paulista, São Paulo, Brazil; 2 Department of Morphology and Pathology, Univ Federal de São Carlos (UFSCar), São Carlos, Brazil; 3 Department of Biostatistics, Institute of Biosciences of Botucatu (IBB), UNESP – Univ Estadual Paulista, São Paulo, Brazil

**Keywords:** alcohol, puberty, preconception, reproduction, litter size, offspring.

## Abstract

**Graphical abstract:**

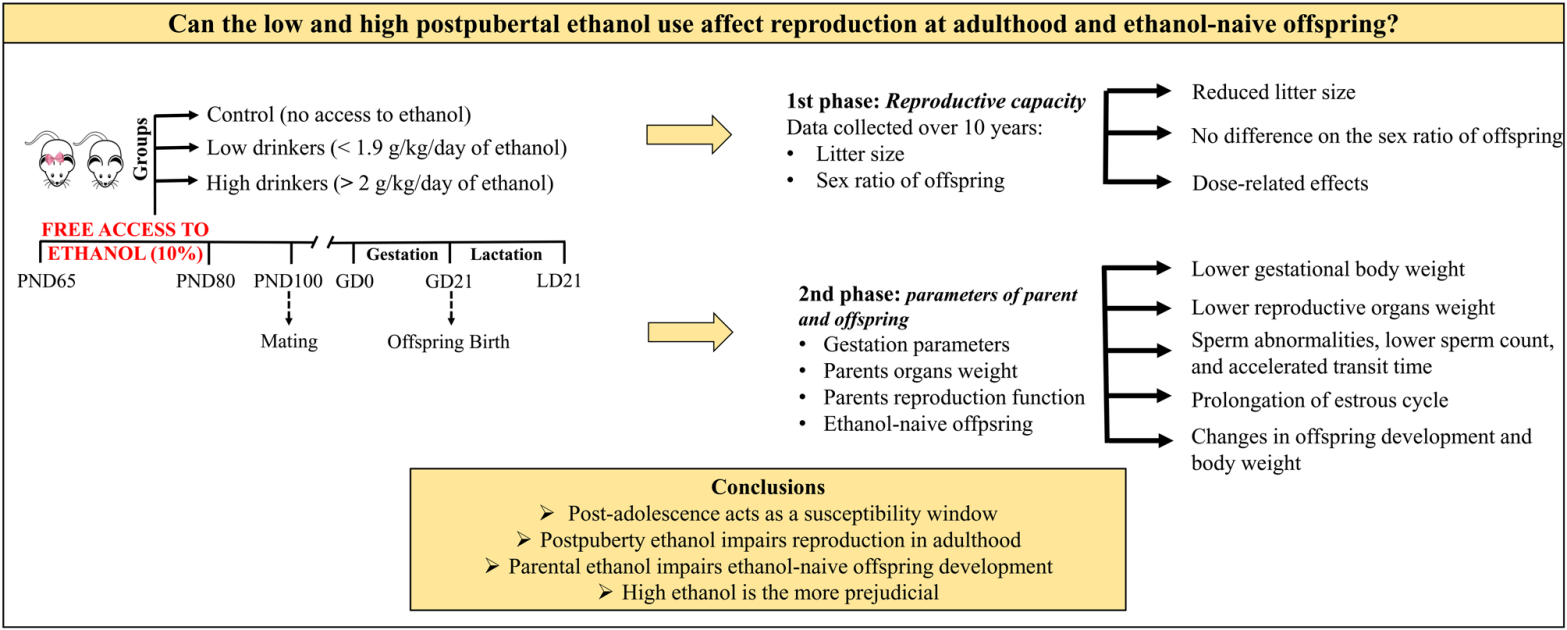

**Abstract:**

The relationship between adolescent ethanol uses and its impacts throughout life are not conclusive. Thus, we evaluated if the low and high consumption of ethanol at postpuberty interferes with the reproduction and ethanol-naive offspring and if the effects are dose-related. Female and male rats were divided into three groups: low drinker (L), high drinker (H) and control (C). The L and H groups were exposed to ethanol up to 10 % from 65 to 80 days with withdrawal after this period. The ethanol consumed by low drinkers was 1.41 ± 0.21 g/kg/day and by high drinkers 4.59 ± 0.45 g/kg/day. The study was conducted in two phases. The first phase verified the reproductive capacity in adulthood on generations (litter size and sex ratio). Data were collected over 10 years. The second phase analyzed the parent reproductive parameters (body weight, reproductive organ weight, sperm parameters and estrous cycle) and the pup development. We observed a reduced litter size in both drinker groups. Gestational body weight gain and feed consumption were lower in L and H. We observed an alteration in reproductive organs weight in both sexes of H. Females presented a longer estrous cycle duration. Males presented an increase in abnormal sperm, a decrease in sperm count and accelerated transit time. The ethanol-naive offspring development was also impaired. We conclude that low and high postpubertal alcohol use impairs long-term reproductive parameters, even after alcohol withdrawal. There is also impaired ethanol-naive offspring. Besides, the effects are dose-related.

**Lay summary:**

The effects of alcohol use have been reported in several studies. However, better knowledge about early alcohol use and its impact on reproduction in adulthood, after abstinence and on ethanol-naive offspring could help improve preventive measures and mechanisms of action. One of the methods used was retrospective analysis which allows to evaluate the effects of postpubertal ethanol use on the reproductive capacity of rats over generations. Despite our limitations, we verified that the post-adolescent period acts as a susceptibility window, and lifestyle at this age modulates the long-term reproductive parameters. The early ethanol use impairs reproduction function since sperm parameters and the estrous cycle have been altered. The dose of alcohol also contributes to damage on the drinkers’ reproduction and on the physical development of ethanol-naive offspring. Future studies are necessary to identify the mechanism involved in long-term alcohol use effects, even in withdrawal, as well as ethanol-naive offspring outcomes.

## Introduction

Ethanol is one of the main abuse drugs ingested worldwide (Rehm *et al.* 2009, Balddin *et al.* 2018), and it is responsible for approximately 5.2% of global deaths (GBD 2018). Alcohol use prevails among adolescents and young adults, and it is the leading risk factor for disability among those aged 15–49 years, accounting for 10% of global deaths in this age group (Harding *et al.* 2016, WHO 2018). The implications of consumption must consider the age, the amount ingested and the consumers’ characteristics (Balddin *et al.* 2018).

Approximately 15% of couples show signs of infertility (Sharlip *et al.* 2002, Barazani *et al.* 2014), being the daily habits, including diet and exposure to toxicants responsible for modulating reproductive health (Asimes *et al.* 2018). Ethanol is a toxic agent that disturbs not only the integrity of biochemical and physiological functions but also the development of structures involved in reproduction, causing severe damage to the signaling of hypothalamic–pituitary–gonadal/adrenal axes (HPG/HPA) (Wallock-Montelius *et al.* 2007). Therefore, alcohol intake can result in female and male reproductive pathologies confirmed in experimental models (Oremosu & Akang 2015, Srivastava *et al.* 2018) and humans (Eggert *et al.* 2004, Sansone *et al.* 2018) since ethanol could lead to lower sperm quality and ovulatory irregularities (Sengupta *et al.* 2017). Besides, ethanol-induced epigenetic mechanisms can modify the expression pattern of different tissues on drinkers, as well as it is the major mechanism related to descendants’ phenotype alterations (Asimes *et al.* 2017).

Studies highlight that heavy drinking during gestation can reduce litter size, increase the mortality rate and impair the offspring; however, the results are inconclusive regarding the sex ratio (Anderson *et al.* 1978, Cicero *et al.* 1994, Vaglenova & Petkov 1998, Gardebjer *et al.* 2014, Liang *et al.* 2014). Although heavy drinking has greater effects on reproduction, low to moderate intake has been still under discussion, requiring constant research (Barazani *et al.* 2014, Sansone *et al.* 2018). Rodent’s prepubertal and preconception ethanol exposure can also be harmful to drinkers and their pups. Nevertheless, there is no evidence that it occurs in the postpuberty period. Thus, studies that aim to verify postpubertal ethanol and its effects on reproduction can help to elucidate the degree of damage of early ethanol intake and the mechanisms involved in this process. The UCh rats, which are voluntary ethanol-drinking models derived from original Wistar rats (Mardones & Segovia-Riquelme 1983), were used. This strain represents a special model to understand the basis of alcoholism-linked characteristics. We hypothesized that the high and low ethanol drinking during postpuberty negatively influences the parameters of reproduction in adulthood, even after ethanol withdrawal, and affects the ethanol-naive offspring with dose-related effects. Therefore, we evaluated whether the low and high ethanol impairs reproductive capacity, function, organ weight of the animal early exposure and the ethanol-naive offspring development. Part of this study was carried out by a collection of data over 10 years, allowing us to assess different generations.

## Materials and methods

### Animals and experimental design

The experiments were in accordance with the Ethical Principles in Animal Research and approved by the Bioscience Institute/UNESP Ethical Committee for Animal Research (protocol nº 051/04). Female (171 ± 6.3 g) and male (231 ± 10.7 g) rats (*Rattus norvegicus albinus*) at 55 days old were obtained from the Department of Structural and Functional Biology of Botucatu Bioscience Institute/UNESP. The animals were housed in polypropylene cages (32 cm × 40 cm × 18 cm) and maintained under controlled conditions (25 ± 1°C, humidity 55 ± 5%, and light from 6 to 18 h) with access to commercial feed and water *ad libitum*. We employed a voluntary model for ethanol exposure, UCh rat strain, avoiding the stress associated with forced feeding and providing knowledge about the effects of voluntary ethanol consumption as observed in society (Gapp *et al.* 2014, Martinez *et al.* 2016).

The rats were divided into three groups: low drinker (L) constituted by UChA rat strain, high drinker (H) constituted by UChB rat strain and control rats (C) without access to ethanol. The ethanol-drinking groups, L and H, were exposed to ethanol for 15 consecutive days for voluntary consumption, period referring to the selection of drinker animals (Mardones & Segovia-Riquelme 1983). Thus, the rats were offered free access to a bottle containing ethanol up to 10% from the postnatal day (PND) 65 to 80, corresponding to postpuberty (Picut *et al.* 2015). The low drinker rats (UChA strain) should drink from 0.1 to 1.9 g/kg/day of ethanol and high drinkers (UChB strain) should drink more than 2.0 g/kg/day. Only rats which drank the stipulated ethanol consumption (low and high ethanol drinkers) were selected to continue in the experiment (Mardones & Segovia-Riquelmi 1983). The ethanol consumption was calculated by consumed ethanol (mL)/15 × 100)/body weight (g). The animals were maintained in individual cages during the period of free access to ethanol for consumption measurement. Female average consumption was 1.56 ± 0.25 g/kg/day for L and 4.90 ± 1.89 g/kg/day for H and the male average consumption was 1.27 ± 0.34 g/kg/day for L and 4.27 ± 1.53 g/kg/day for H. The average total ethanol consumption during the 15 days of exposure was 78.14 mL ± 82.52 for L females and 176.30 mL ± 95.52 for H females and 91.45 mL ± 97.15 for L males and 205.69 mL ± 106.47 for H males.

**Figure 1:**
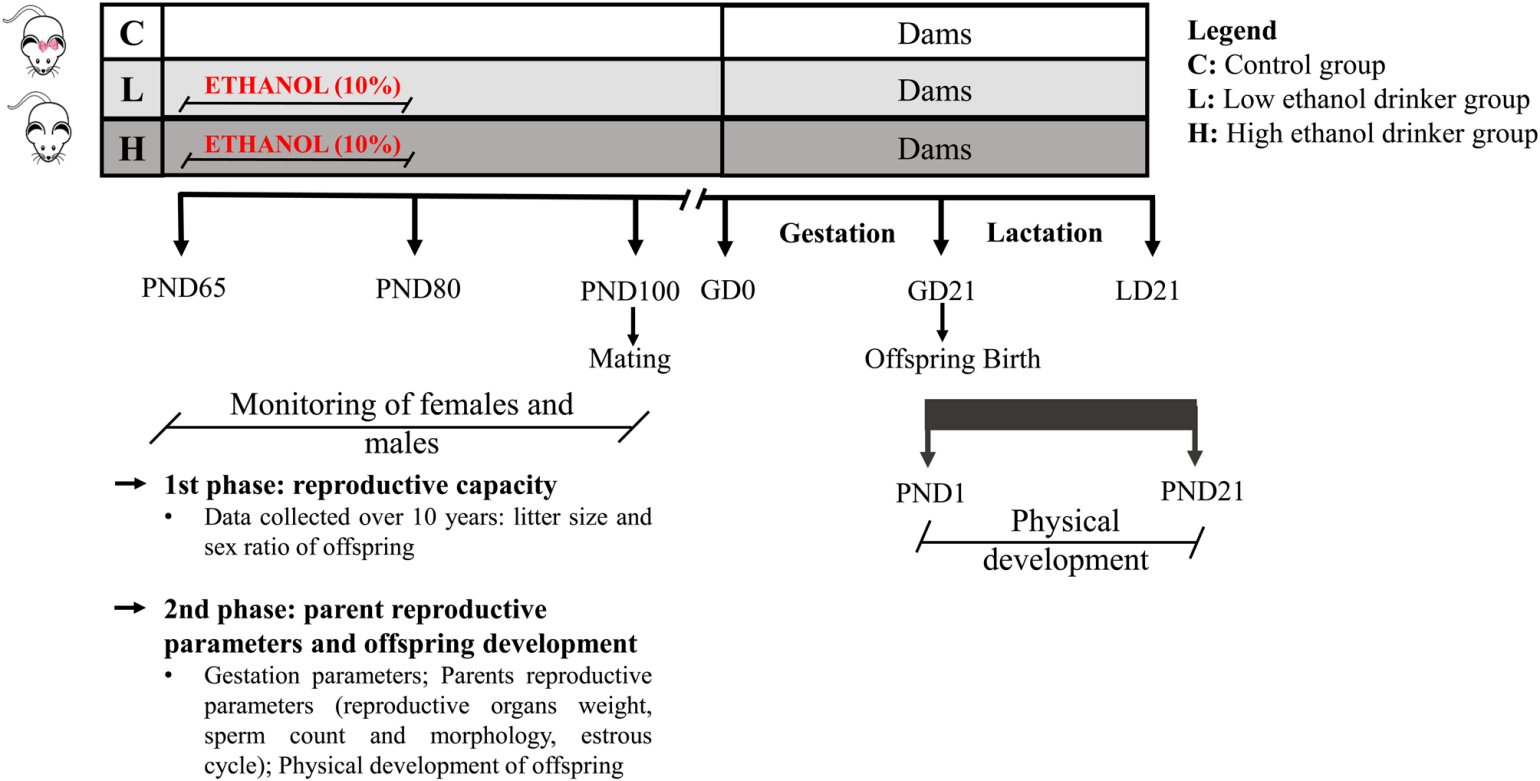
Diagram of the experimental design. GD, gestational day; LD: lactational day; PND, postnatal day. Female and male rats were divided into three groups: low drinker (L), with rats drinking 1.41 ± 0.21 g/ kg/day, high drinker (H), with rats drinking 4.59 ± 0.45 g/ kg/day and control (C), with rats without access to ethanol. The ethanol-drinking groups, L and H, were exposed to ethanol for 15 consecutive days for voluntary consumption. Thus, a bottle containing ethanol up to 10% was offered to rats to free access from the postnatal day (PND) 65 to 80, corresponding to postpuberty. The ethanol was withdrawn after this period to mating posteriorly. The study was conducted in two phases. The retrospective analysis, first phase, verified the reproductive capacity. The data were collected over 10 years to verify litter size and the sex ratio of offspring from C, L and H groups. The second phase verified body weight and feed consumption at gestation, maternal and paternal reproductive parameters and physical development of offspring from C and H groups.

The ethanol bottle was withdrawn after 15 days of free access to allow mating in order to ensure that the observed effects were from postpubertal ethanol consumption. Females of C, L and H groups were mated to males of C, L and H, respectively, at 100 days old, age deemed sexually mature. This study was conducted in two experimental phases (Fig. 1). The first one utilized the retrospective analysis to verify litter size and sex ratio of offspring from control and from low and high ethanol drinkers’ groups over generations. The data were collected over 10 years (2005–2015) at Anatomy’s Bioterium (IBB/UNESP) to analyze the postpuberty ethanol use effects on adulthood reproductive capacity. Due to significant results from the first phase, we additionally analyzed the gestational parameters (body weight and feed and water consumption), maternal and paternal reproductive parameters (reproductive organs weight, sperm count and morphology and estrous cycle) and the initial development of ethanol-naive offspring (landmarks of physical development and body weight) in the second phase (Fig. 1).

### First experimental phase reproductive capacity

#### Litter size and sex ratio of offspring

The count of female and male pups per dam at offspring birth (PND 0) was realized. The litter size and sex ratio of offspring were analyzed from C, L and H (*n*  = 110 litters/group). The sex ratio of offspring was verified by the count of females and males at birth since sex dimorphism in neonates is evidenced by the shorter distance between the anus and the genital tubercle of females (Gallavan *et al.* 1999). Only the first generation (F1) data were considered in this analysis. The long-term reproductive capacity was determined by the litter size from the first and second-generation (F1 and F2, *n*  = 15 couples/generation/group).

The data about litter size and offspring sex ratio were collected over 10 years.

### Second experimental phase parent reproductive parameters and offspring development

#### Dam parameters on gestation

In order to evaluate the evolution of pregnancy, females of C (*n*  = 8), L (*n* =  8) and H (*n*  = 8) were mated to males of C (*n*  = 8), L (*n*  = 8) and H (*n*  = 8), respectively, at 100 days old at overnight (one female and one male/cage). A vaginal smear was carried out daily in the morning, and the first day of pregnancy was considered when spermatozoa were found. After the pregnancy detection, gestational day (GD) 0, the dams were individualized and monitored. Body weight and feed and water consumption during gestation were measured weekly and weighed on an analytical balance.

### Parents reproductive organs weight and adiposity index

The females (*n*  = 8/group) and males (*n*  = 8/group) from control (C) and high ethanol drinker (H) were weighed and euthanized by CO_2_ inhalation followed by decapitation. Males were killed at PND 150 while females were killed from the PND 150 in the estrous phase. The testis, epididymis, ventral prostate and seminal vesicle (with fluid) in the males and ovaries and uterus in the females were removed, dissected and weighed on an analytical balance. The relative organs weight was calculated by organ weight (mg)/body weight (g). The adiposity index was also calculated by [(retroperitoneal fat + visceral fat + epididymal/ovarian fat)/final body weight] × 100.

### Maternal estrous cycle

The estrous cycle from C and H females was assessed based on vaginal smears collected every morning for 10 days from PND 140. The samples were analyzed under a light microscope, and estrous cycle phases were classified as metestrus (leukocytes and cornified and nucleated epithelial cells), diestrus (leukocytes), proestrus (nucleated epithelial cells) and estrus (anucleate cornified cells) (Marcondes *et al.* 2002). The estrous cycle duration was calculated by the number of days between one estrous phase to the next and the number of estrous cycles during the assay (Borges *et al.* 2017).

### Paternal sperm count, daily sperm production and epididymal transit time

Sperm count was performed in C and H males. Homogenization-resistant testicular spermatids and sperm in the caput/corpus and cauda epididymal were obtained from testis and epididymis (left side) and were counted as described by Robb *et al.* (1978). The sperm count was determined using the Neubauer chambers. Two Neubauer chambers, divided into 2 antimeres, were prepared per animal, accounting for 20 fields/animal. Spermatid numbers were obtained by sperm count mean multiplied by the dilution factor. Sperm concentration (spermatids/g testis) was obtained by the spermatid counts mean divided by the weight of testicular parenchyma. Daily sperm production was obtained dividing the total number of homogenization-resistant spermatids per testis by 6.1, the number of days in which these spermatids are present on germinative epithelium (Robb *et al.* 1978). Transit time through the caput/corpus and cauda epididymis was calculated dividing the number of sperm within each of these regions by the daily sperm production (Robb *et al.* 1978).

### Paternal sperm morphology

The sperm from C and H groups were obtained by a wash of vas deferens with a PBS solution. The volume of 10 µL was obtained from vas deferens, allocated on Eppendorf and maintained on a refrigerator (20°C) until analyzed. Spermatic fluid was placed on the slide, dried at room temperature for 10 min and evaluated under phase-contrast microscopy (400×, total magnification). Two hundred sperm per animal were evaluated for head or flagellar defects (Seed *et al.* 1996). Anomalies were classified into head anomalies (neither typical nor isolated hook) or tail anomalies (broken or tail headless), and the data were expressed in percentage (Filler 1993).

### Offspring body weight and landmarks of physical development

At birth, the offspring were cut to eight pups (four females and four males) per dam. The body weight of offspring was measured on birth from C, L and H (*n*  = 32 sex/group) and the litter body weight (*n*  = 8/litter/group) was weekly monitored, from PND 1 to 21, the period that includes the neonatal (PND 0–7), early infantile (PND 8–14) and late infantile (PND 15–21) phases. The pups were weighed on an analytical balance. To evaluate the initial physical development, pinna unfolding, hair growth and eye-opening were also daily observed.

### Statistical analysis

The data were analyzed by the software GraphPad Prism® (version 7, GraphPad Software). A one-way ANOVA (parametric data) was used in physical development of offspring. *Post hoc* analysis was performed by Tukey’s multiple comparison test. A Kruskal–Wallis (non-parametric data) test was used in determining litter size and offspring sex ratio. *Post hoc* analysis was performed by multiple comparison Dunn’s test. A two-way ANOVA was employed in dams’ parameters on gestation and offspring body weight gain. *Post hoc* analysis was performed by Sidak’s multiple comparison test. Time, treatment and interaction values were expressed in the figure and table legends. Unpaired *t*-test (parametric data) was employed in parent reproductive parameters. Results were expressed as mean ± s.d. or median and interquartile range. The differences were considered significant when *P* < 0.05.

## Results

### First experimental phase reproductive capacity

#### Postpuberty ethanol uses reduced litter size with dose-related effects but did not impact the sex ratio of offspring

**Figure 2:**
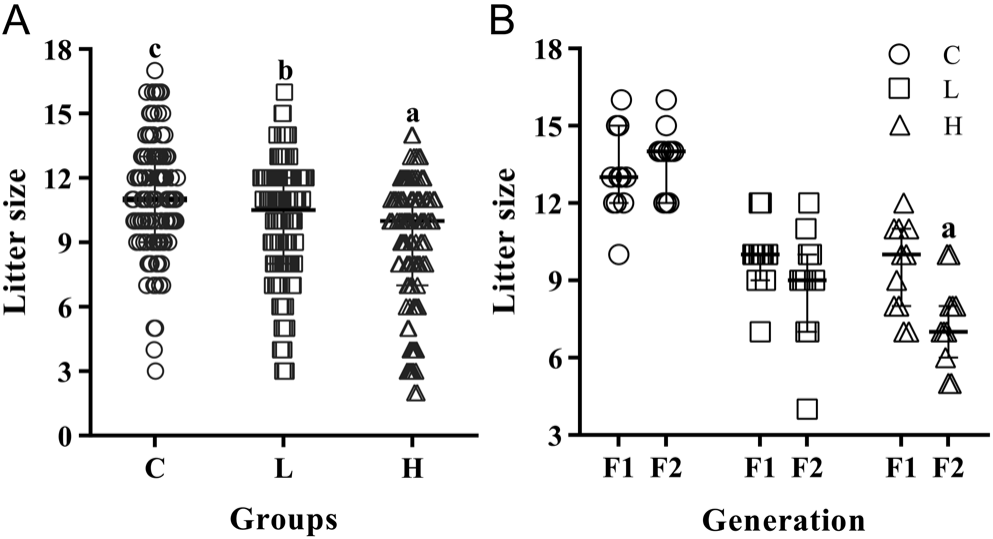
Comparison of litter size from control (C), low drinker (L) and high drinker (H) groups. (A) Litter size from C (*n*  = 110), L (*n*  = 110) and H (*n*  = 110) groups. Values expressed as median and interquartile range. *P*-values were calculated using a Kruskal–Wallis test. ^a, b, c^Different letters represent significant differences among groups (*P* < 0.05) from* post hoc* Dunn’s multiple comparisons test. (B) Litter size of first (F1) and second (F2) generation from C (*n*  = 15), L (*n*  = 15) and H (*n*  = 15) groups. Values expressed as mean ± s.d.*P*-values were calculated using a two-way ANOVA. ^a^Significant difference between generations (*P* < 0.05) from* post hoc* Sidak’s multiple comparison test. Figure 1B: *P*_Inter_ = 0.0595, *P*_Time_ = 0.0709, *P*_Treat_ < 0.0001.

The litter size from L and H groups was lower compared to C. We observed reduced litter size in the H (Fig. 2A) between drinkers’ groups. Thus, the greater ethanol use was the most damaging to the litter size. The comparison of litter size among generations is represented in Fig. 2B. Only the H group showed reduced litter size comparing F1 to the F2.

There were no differences in the sex ratio of offspring from C, L and H groups (females: C = 51.38 % ± 15.82; L = 50.53 % ± 18.02; H = 50.74 % ± 17.30; males: C = 48.62 % ± 15.53; L = 49.47 % ± 18.02; H = 49.26 % ± 17.00).

### Second experimental phase parent reproductive parameters and offspring development

#### Low and high postpuberty ethanol use decreased gestational body weight gain and feed consumption, no dose-related effects

**Figure 3:**
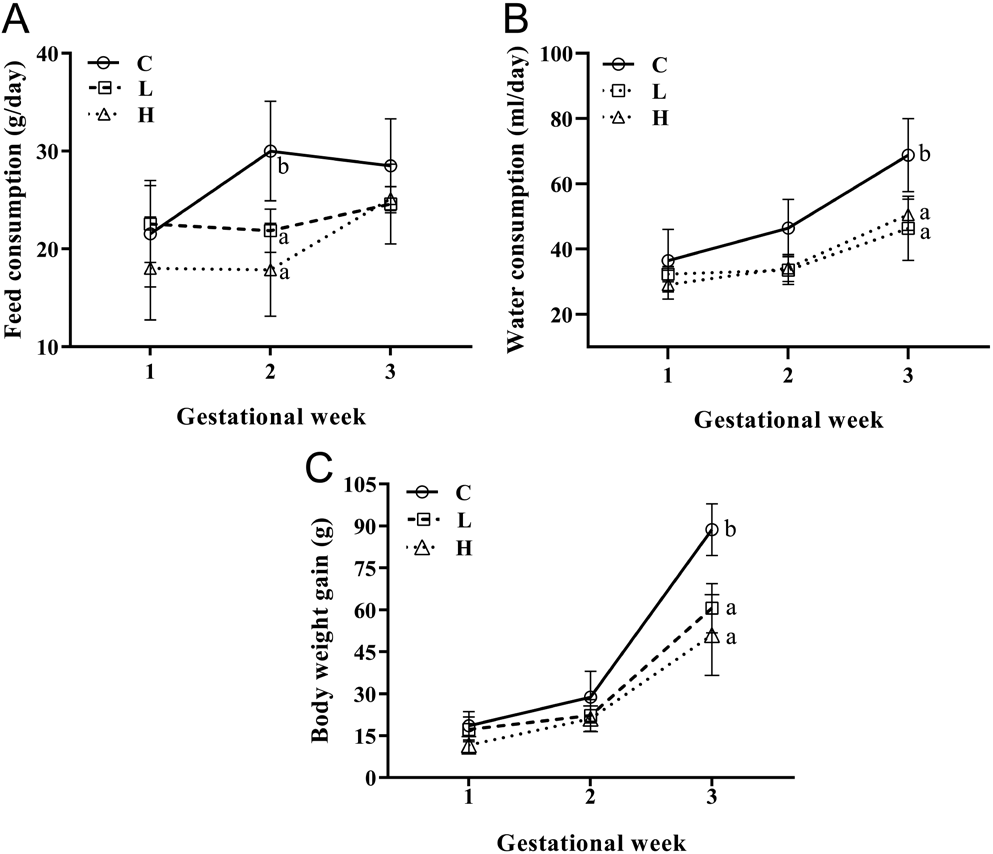
Dams’ parameters on gestation of control (C), low drinker (L) and high drinker (H) groups. (A) Feed consumption, (B) water consumption, (C) body weight gain of C (*n*  = 8), L (*n*  = 8) and H (*n*  = 8). Values expressed as mean ± s.d. *P*-values were calculated using a two-way ANOVA. ^a, b^Different letters represent significant differences among groups (*P*  < 0.05) from* post hoc* Sidak’s multiple comparison test. Figures 2A: *P*_Inter_ = 0.0122, *P*_Time_ < 0.0008, *P*_Treat_ < 0.0001; 2B: *P*_Inter_ = 0.0701, *P*_Time_ < 0.0001, *P*_Treat_ < 0.0001; 2C: *P*_Inter_ < 0.0001, *P*_Time_ < 0.0001, *P*_Treat_ < 0.0001.

The maternal body weight on GD 0 and GD 21 was lower in both postpubertal ethanol exposed groups compared to control (GD0 C: 285.4 g ± 14.9, L: 236.7 g ± 11.5, H: 246.4 g ± 11.8; GD 21 C: 315.0 g ± 34.7, L: 286.7 g ± 12.7, H: 287.2 g ± 7.8). However, the dams body weight gain from L and H groups was lower only on the third gestational week (Fig. 3C). Regarding the feed and water consumption, we observed lower consumption in the L and H dams on the second and third gestational weeks, respectively (Fig. 3A and B).

### High postpubertal ethanol use impaired parent reproductive organs weight on adulthood and compromised the reproductive function

In this analysis, only data from the control and high drinker groups were compared due to insufficient data from the low drinker group. Regarding female reproduction function, we observed a greater estrous cycle duration in females previously exposed to ethanol (C: 4.6 ± 0.4; H: 5.1 ± 0.4). There was no change in the sequence of the estrous cycle classified as metestrus, diestrus, proestrus and estrus. On the other hand, 62.5% of the H group had prolonged estrous and proestrous phases for 2 days. There was lower absolute and relative uterine weight and adiposity index in the females from the H group, but the body weight did not alter (Table 1).

**Table 1:** Comparison of body weight, relative and absolute reproductive organs weight and adiposity index at postnatal day 150 in the females and males from control (C) and high drinkers (H) groups (*n*  = 8/sex/group). Values expressed as mean ± s.d.

Parameters	Groups	
	C	H
Females		
Body weight (g)	315.01 ± 30.99	287.2 ± 8.77
Ovaries (g)	0.30 ± 0.06	0.25 ± 0.01
Ovaries (mg/g)	0.46 ± 0.04	0.43 ± 0.02
Uterus (g)	0.67 ± 0.61	0.46 ± 0.04*
Uterus (mg/g)	2.13 ± 0.12	1.62 ± 0.15*
Adiposity index	3.99 ± 0.73	3.00 ± 0.31*
Males		
Body weight (g)	506.05 ± 12.22	408.00 ± 16.37*
Testis (g)	3.71 ± 0.28	3.27 ± 0.21*
Testis (mg/g)	3.67 ± 0.29	4.02 ± 0.29
Epididymis (g)	1.56 ± 0.05	1.40 ± 0.12*
Epididymis (mg/g)	1.50 ± 0.09	1.72 ± 0.02*
Ventral prostate (g)	1.56 ± 0.12	1.34 ± 0.27
Ventral prostate (mg/g)	3.08 ± 0.22	3.28 ± 0.67
Seminal vesicle (g)	2.01 ± 0.50	2.30 ± 0.34
Seminal vesicle (mg/g)	3.95 ± 0.95	5.62 ± 0.77*
Adiposity index	2.93 ± 0.62	2.85 ± 0.46

*P*-values were calculated using a *t*-test.

*Significant difference between groups (*P* < 0.05).

In the males, we verified lower body weight and absolute testis and epididymis weight while the relative epididymis and seminal vesicle weight were greater on H (Table 1). The sperm parameters were also altered in males exposed early to ethanol. There was a decrease in sperm count in the cauda epididymal and acceleration of total sperm transit time (Table 2). Furthermore, an increase in the percentage of sperm with morphologic abnormalities was observed, including a higher incidence of head and tail defects (Table 3).

**Table 2:** Paternal sperm count, daily sperm production and epididymal transit time at postnatal day 150 in the males from control (C) and high drinkers (H) groups (*n*  = 8/group). Values expressed as mean ± s.d.

Parameters	Groups	
	C	H
Testis		
Spermatid number (×10^6^ /g/day)	86.69 ± 11.33	97.27 ± 21.77
Daily sperm production (×10^6^//testis/day)	14.21 ± 1.85	15.95 ± 3.57
Caput/corpus epididymal		
Sperm number (×10^6^/g/organ)	235.4 ± 45.87	198.30 ± 43.49
Sperm transit time (days)	3.91 ± 0.77	3.62 ± 1.25
Cauda epididymal		
Sperm number (×10^6^/g /organ)	698.6 ± 139.10	539.4 ± 116.30*
Sperm transit time (days)	7.73 ± 1.85	5.29 ± 2.96
Total sperm transit time (days)	12.24 ± 1.83	9.13 ± 2.08*

*P*-values were calculated using a *t*-test.

*Significant difference between groups (*P* < 0.05).

**Table 3:** Paternal sperm morphology at postnatal day 150 in the males from control (C) and high drinkers (H) groups (*n*  = 8/group).

Parameters (%)	Groups	
	C	H
Normal sperm	91.25 (90.38–92.63)	75 (70.38–81.25)*
Abnormal sperm	8.75 (7.37–9.62)	25 (18.75–29.63)*
Broken tail	0.56 (0.50–0.71)	0.75 (0.00–3.00)
Headless	1.56 (0.87–2.09)	4.00 (2.87–4.12)*
Isolated head	5.87 (4.50–6.87)	19.75 (12.50–23.75)*

Values expressed as median and interquartile range (IQ1–IQ3). *P*-values were calculated using a *t*-test.

*Significant difference between groups (*P* < 0.05).

#### Low and high postpubertal parental ethanol use impaired body weight and physical development of ethanol-naive offspring, with dose-related effects

**Figure 4:**
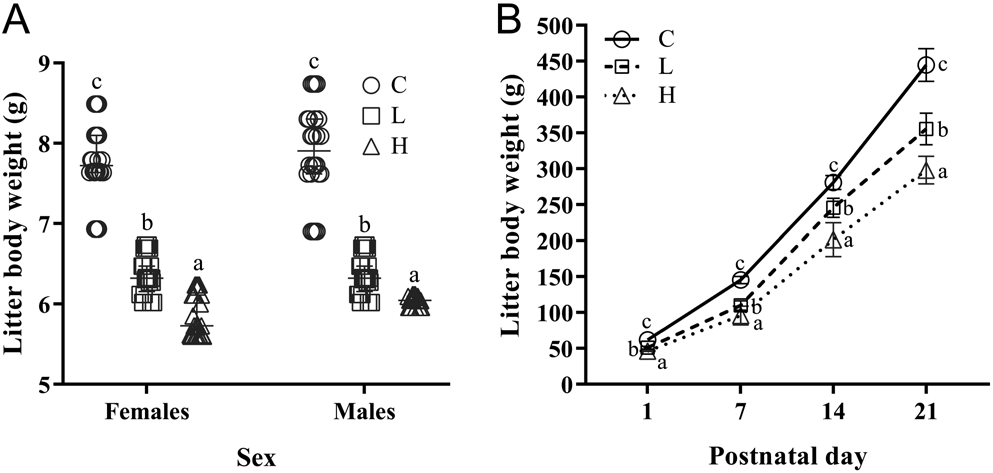
Parameters of female and male offspring from control (C), low drinker (L) and high drinker (H) groups. (A) Body weight on birth of females and males (*n*  = 32/sex/group). Values expressed as median and interquartile range. *P*-values were calculated using a Kruskal–Wallis test. ^a, b, c^Different letters represent significant differences among groups (*P* < 0.05) from* post hoc* Dunn’s multiple comparisons test. (B) Litter body weight (females and males) on postnatal day 1–21 (*n*  = 8 /litter/ group). Values expressed as mean ± s.d.*P*-values were calculated using a two-way ANOVA. ^a, b, c^Different letters represent significant differences among groups (*P* < 0.05) from* post hoc* Sidak’s multiple comparison test. Figure 3B: *P*_Inter_ < 0.0001, *P*_Time_ < 0.0001, *P*_Treat_ < 0.0001.

Figure 4 represents the parameters of female and male offspring from C, L and H groups. The pups sired by low and high postpubertal parental ethanol use had a lower body weight at birth and throughout the infant period (PND 1–21) compared to control (Fig. 4A). The damages on the offspring were correlated to the amount of ethanol consumed by parents since the offspring from the high drinker group showed lower body weight compared to offspring from the low drinker group.

The landmarks of physical development were changed on the offspring from low and high drinker groups. Earlier eye-opening in the offspring from L and delayed hair growth in the H was observed (Table 4).

**Table 4:** Comparison of the mean day of physical development landmarks in days in the offspring (*n*  = 8/litter/group) from control (C), low drinker (L) and high drinker (H) groups.

Parameters (days)	Groups		
	C	L	H
Pinna unfolding	2.5 ± 0.5	2.3 ± 0.8	3.1 ± 0.8
Hair growth	4.7 ± 0.4^a^	4.8 ± 0.5^a^	5.7 ± 0.4^b^
Eye-opening	14.2 ± 0.3^b^	13.6 ± 0.3^a^	14.3 ± 0.5^b^

Values expressed as mean ± S.D. *P*-values were calculated using a one-way ANOVA.

^a, b^Different letters represent significant differences among groups (*P* < 0.05) from* post hoc* Tukey’s multiple comparison test.

## Discussion

This is the first study to conduct a retrospective analysis of postpubertal ethanol use and its effects on the reproductive capacity in the voluntary model of ethanol consumption. The collection of data over 10 years highlights that low and high postpuberty ethanol use impairs reproductive capacity in adulthood, even after alcohol withdrawal. The reproductive function of males and females exposed early to ethanol was altered in adulthood as we observed changes in weight of reproductive organs in both sexes, a greater estrous cycle duration in females, and a decrease in sperm parameters in males. The landmarks of physical development and body weight of ethanol-naive offspring were also impaired. Taken together, our data indicate lifestyle after adolescence, such as ethanol use, could modulate long-term reproduction, even after ethanol withdrawal, and future generations.

Postpubertal alcohol use led to lower weight gain and feed consumption on gestation in the L and H dams, with dose-related effects. Increased plasma leptin during abstinence (Kiefer *et al.* 2005) contributes to decreased food intake. On the other hand, the inefficiency in the absorption of ingested calories can also act since the metabolism of ethanol changes the intestinal permeability and harms the organic systems function (Bishehsari *et al.* 2017). In addition, the lower weight gain could be associated with the weight of the pregnant uterus (Kind *et al.* 2006), fetal and placental (Brett *et al.* 2014), as we found lower litter size and body weight at birth of the offspring from L and H groups.

Considering the direct-ethanol toxicity, gestational exposure can reduce litter size, especially in the high drinkers (Cicero *et al.* 1994, Ojeda *et al.* 2009, Li *et al.* 2013). Interestingly, we found that ethanol consumption only on postpuberty also decreased litter size but did not alter offspring sex ratio. Impacts on blastocyst implantation, oxidative damage to germline DNA compromising the embryo cells, abnormal fetal development and increased rates of resorption and abortion may be mechanisms contributing to these results (Cicero *et al.* 1994, Emanuele *et al.* 2001*b*, Jana *et al.* 2010, Jensen *et al.* 2014). We hypothesized that the reproductive capacity of high drinkers was chronically affected, as there was a decreased litter size between the first and second generation.

In this perspective, we observed that besides the impairment on the gestational parameters of dams early exposed to ethanol, the offspring also showed reduced body weight, with dose-related effects. Studies highlight that preconception ethanol exposure can influence the descendants’ phenotype. Reduction in the gestational sac weight and placental efficiency and function due to maternal and paternal ethanol use (Chang *et al.* 2017, Gardebjer *et al.* 2014) could explain our results partially. Preconception nutrition, body weight index, gestational weight gain and food consRobertson 2005, Rando & Simmons 2015) since the seminal fluid stimulates the female reproductive tract to produce growth factors and cytokines which protect the embryo (Robertson 2005), and changes in the seminal signalization can also influence descendants (Bromfield *et al.* 2014). The landmarks of physical development of offspring sired by alcoholic parents were altered in our study, similar to results by Fioravante *et al.* (2021). The insulin-like growth factor (IGF) is important for fetal and postnatal development, and IGF deficiency implicates signaling pathways and normal body growth (Kanaka-Gantenbein *et al.* 2003). Similarly, the EGF plays a role in regulating the activity of epidermal and epithelial tissues such as eye-opening and hair growth (Calamandrei & Alleva 1989, Smart *et al.* 1989). The alterations in the postnatal development of offspring can be also associated with maternal care (Amorim *et al.* 2011) since alcohol withdrawal accentuates depressive behaviors and reduced time spent on nursing (Pang *et al.* 2013, Workman *et al.* 2015). We hypothesized a possible endocrine and metabolic programming of the offspring, with the parental ethanol dose as decisive in the course of this programming.

Regarding females and males exposed to high ethanol, we found impairment in reproductive function and parameters in both sexes. The reproductive organs’ weight has been used to evaluate the toxicity of the reproductive system (Clegg *et al.* 2001). In this perspective, we found lower absolute and relative uterine weight and prolonged estrous cycle in H females. The ovary can often respond to cyclic alterations promoting a constant estrus as verified by Krueger *et al.* (1983) who observed a disruption in the estrous cycle by alcohol use. Our previous laboratory studies also verified a reduction of luteinizing hormone and follicle-stimulating hormone, follicular atresia, and damage in uterine endometrial cells in drinker females of UCh strain, with dose-related effects; however, ovulation and luteogenesis were present (Chuffa *et al.* 2009, Martinez *et al.* 2016). The increase of acetaldehyde and oxidative stress by ethanol impairs HPG/HPA axis, and they are mechanisms that change reproductive hormones balance and, consequently, uterine and ovarian tissues (Buthet *et al.* 2013, Rachdaoui & Sarkar 2017). We suggested that lower uterine weight in H females could be related to a hormonal imbalance with damage to uterine structure and function while estrous prolongation could be associated with estradiol disbalance. Although we did not analyze this hormone to corroborate this hypothesis, studies already observed an alteration in their levels (Emanuele *et al.* 2001*a*, Chuffa et al 2009). The lower adiposity index observed in females from the H group could also highlight possible malnutrition related to loss of muscle or fat mass (Dasarathy 2016).

Relating to male reproductive parameters, studies have verified that ethanol exerts a direct effect on both testosterone metabolism and spermatogenesis (Sansone *et al.* 2018). In contrast to the literature that reports atrophy of reproductive organs in drinkers (Martinez *et al.* 2000, 2001), we found an increase in the epididymis and seminal vesicle relative weight. This finding could be partially associated with lower body weight on H males since it is necessary to use the body weight to calculate the relative weight. Besides, no difference was noticed in the absolute seminal vesicle and there was a decrease in testis and epididymis absolute weight in the H group, corroborating our hypothesis. Analysis of body weight carries out information on the general toxicity of a substance and its possible implications for health (Fernandez *et al.* 2008) and could indicate estrogen imbalance (Heywood & Wadsworth 1980, Hart 1990). The lower body weight on H highlights a possible systemic compromise. Regarding reproductive function, we found decreased sperm count on epididymis and accelerated total transit time. Sperm transit time has an important role in the maturation of spermatozoa, and the acceleration of sperm transit impairs the necessary time for this process (Klinefelter 2002). Additionally, the lower sperm reserve observed in the epididymis could be explained by the acceleration of transit time throughout this organ. Associated, there was an increased percentage of sperm with morphologic abnormalities similar to clinical (Pajarinen *et al.* 1996, La Vignera *et al.* 2013, Sansone *et al.* 2018) and experimental (Jana *et al.* 2010) studies. These abnormalities can be due to failures either in the spermatogenic process or in sperm maturation due to acceleration of transit time. Inadequate signalization of epididymal factors which plays a role in maturation or low testosterone levels can also drive to this (Koch *et al.* 2015, Zi *et al.* 2015). The abnormal testosterone/estradiol ratio has been also associated with decreased semen parameters as well as harm to accessory sex glands (Schulster *et al.* 2016). Taken together, we hypothesized that the quality of the spermatozoa was harmed, reducing fertility potential since damage to reproductive organs and testosterone had already been observed even on alcoholic withdrawal (Candido *et al.* 2007). Although additional analysis is needed to validate the real harm of ethanol, our data indicate that early high alcohol use can impair reproductive function in both sexes. Low doses are also harmful; nevertheless, their impacts are lesser than the high doses (Patra *et al.* 2011, Rahimipour *et al.* 2013).

In summary, the results presented here highlight the alarming possibility that exposure to ethanol during postpuberty produces long-term effects on adulthood reproductive capacity, even in ethanol withdrawal. The ethanol consumption decreased body weight, gestational feed intake and litter size. In addition, there were impairments on reproductive function as well as altered reproductive organs weights in females and males exposed to ethanol early. Besides the impairments on consumers, the offspring development and growth were also affected. However, we cannot distinguish which parent contributed the most to observed changes. However, our previous laboratory studies along with published data strongly suggest maternal influence as the main factor. The reproductive capacity (litter size) of parents and body weight and physical development of ethanol-naive offspring show a dose–effect relationship.

Despite our limitations, we highly believe that the post-adolescent period acts as a susceptibility window. Future studies are needed to identify the mechanisms involved in long-term effects on drinkers’ reproduction as well as ethanol-naive offspring outcomes. Possibly, the effects are associated with epigenetic germline modifications, metabolism activity and HPG/HPA axis.

## Declaration of interest

The authors declare that there is no conflict of interest that could be perceived as prejudicing the impartiality of the research reported.

## Funding

This study was financed by the Grant 2018/12354-5, São Paulo Research Foundation (FAPESP), and Coordination for the Improvement of Higher Education Personnel – Brazil (CAPES) – Finance Code 001.

## Author contribution statement

V C F conceived the study, performed experiments, analyzed and finished data and wrote the paper. A R G and V M B C performed experiments. P F F P, M M, C R P and F E M provided training to perform the experiments and intellectual input for the experimental design and data analysis. All authors contributed to editing the paper.
